# Soy Food Consumption Is Inversely Associated with Handgrip Strength: Results from the TCLSIH Cohort Study

**DOI:** 10.3390/nu15020391

**Published:** 2023-01-12

**Authors:** Hongmei Wu, Jing Quan, Xuena Wang, Yeqing Gu, Shunming Zhang, Ge Meng, Qing Zhang, Li Liu, Xing Wang, Shaomei Sun, Qiyu Jia, Kun Song, Jian Huang, Junsheng Huo, Bing Zhang, Gangqiang Ding, Kaijun Niu

**Affiliations:** 1 Nutritional Epidemiology Institute and School of Public Health, Tianjin Medical University, Tianjin 300070, China; 2 School of Public Health, Tianjin University of Traditional Chinese Medicine, Tianjin 301617, China; 3 Tianjin Key Laboratory of Environment, Nutrition and Public Health, Tianjin 300070, China; 4 Center for International Collaborative Research on Environment, Nutrition and Public Health, Tianjin 300070, China; 5 Radiation Epidemiology Research Center, Institute of Radiation Medicine, Chinese Academy of Medical Sciences & Peking Union Medical College, Tianjin 300192, China; 6 Department of Toxicology and Sanitary Chemistry, School of Public Health, Tianjin Medical University, Tianjin 300070, China; 7 Health Management Centre, Tianjin Medical University General Hospital, Tianjin 300052, China; 8 Chinese Center for Disease Control and Prevention National Institute for Nutrition and Health, Beijing 100050, China

**Keywords:** soy food, handgrip strength, diet, epidemiology

## Abstract

Background: Soy foods contain high levels of soy protein or isoflavones, which can stimulate muscle protein synthesis and increase antioxidant capacity, and thus ameliorate muscle strength decline. However, data from epidemiological studies investigating the association of habitual soy food consumption with muscle strength decline among general Chinese adults are limited. Methods: This study included 29,525 participants (mean age: 41.6 years; 16,933 (53.8%) males). Soy food consumption was evaluated using a validated 100-item food frequency questionnaire. Handgrip strength (HGS) was assessed with a hand dynamometer. Analysis of covariance were performed to assess the multivariable-adjusted least square means (LSM) and 95% confidence interval (CI) for HGS. Results: The multiple adjusted LSM (95% CI) of HGS across soy food consumption were 35.5 (34.2, 37.1) kg for <1 time per week, 36.1 (34.6, 37.6) kg for 1 time per week, 36.3 (34.8, 37.8) kg for 2–3 times per week, and 36.6 (35.1, 38.0) kg for ≥4 times per week (*p* for trend < 0.001). Compared to participants with soy food consumption less than one time per week, the multiple adjusted odds ratio (95% CI) of low HGS was 0.638 (0.485, 0.836) when the weekly consumption was ≥ 4 times (*p* for trend < 0.01). Conclusions: Higher habitual soy food consumption was positively associated with HGS in general Chinese adults. Consumption of soy foods may have beneficial effects on muscle health.

## 1. Introduction

Age-related muscle strength decline is a common health problem affecting adults [1,2]. The presence of low muscle strength is a central component of sarcopenia, and the prevalence of sarcopenia continues to increase worldwide [3,4,5]. Moreover, ample evidence has revealed that muscle strength decline can result in a series of adverse health outcomes including cardiovascular disease, mobility limitation and overall mortality [6,7]. As such, it is of great importance to identify potential modifiable lifestyle factors to prevent muscle strength decline. Growing evidence have supported that nutrition plays a key role in the prevention and treatment of muscle strength decline [8,9,10]. Nutritional supplementation, such as high quality protein, essential amino acids, polyunsaturated fatty acids and vitamins, can improve muscle function by stimulating muscle protein synthesis in older adults [11,12,13]. Soy food is traditional food in Asia, especially in China. Soy food consumption is much higher in the Chinese population than in the Western population. Soy food is frequently included in Chinese dietary guidelines. It is cheap, affordable and is uniquely rich in high quality protein, vitamins, polyunsaturated fat and is the primary source of isoflavones [14]. All of these nutrients are essential for the health of skeletal muscle [15,16,17]. For example, in animal studies, Abe T. et al. demonstrated that supplementation of dietary soy glycinin protein could prevent myofiber cross sectional area decrease and muscle atrophy in denervated mice by suppressing ubiquitination and degradation of insulin receptor substrate-1 [18]. Meanwhile, Messina S. et al. showed that soy isoflavone supplementation could ameliorate muscle function and morphology by reducing oxidative stress, inhibiting proinflammatory pathways in mdx mice [19]. In addition, the beneficial effects of soy protein or soy isoflavone on muscle strength have also been reported in many clinical studies [20,21]. For example, a 6-month four-arm randomized controlled trial study of 123 Chinese older adults showed that supplementation with soy protein can prevent muscle mass loss and physical performance decline [22]. Moreover, another meta-analysis conducted by Munguia L et al. found that flavonoids have beneficial effects on skeletal muscle metabolism and mitochondria function, and thereby could be used as a potential tool in preventing skeletal muscle loss [23].

However, until now, though many studies have identified a protective effect of soy components (soy protein or soy isoflavone) on muscle health, it is unknown how habitual consumption of soy foods affects muscle health in the general population. Because soy foods are traditionally consumed daily by Chinese adults, we evaluated whether soy food consumption was associated with a higher HGS in general Chinese adults.

## 2. Methods

### 2.1. Study Population

The study rationale and design of the Tianjin Chronic Low-grade Systemic Inflammation and Health (TCLSIH) Cohort Study have been described in detail in previous published studies [24,25]. Informed consent was obtained from each participant and the protocol was approved by the Institutional Review Board of Tianjin Medical University (reference number: TMUhMEC 201430).

**Figure 1:**
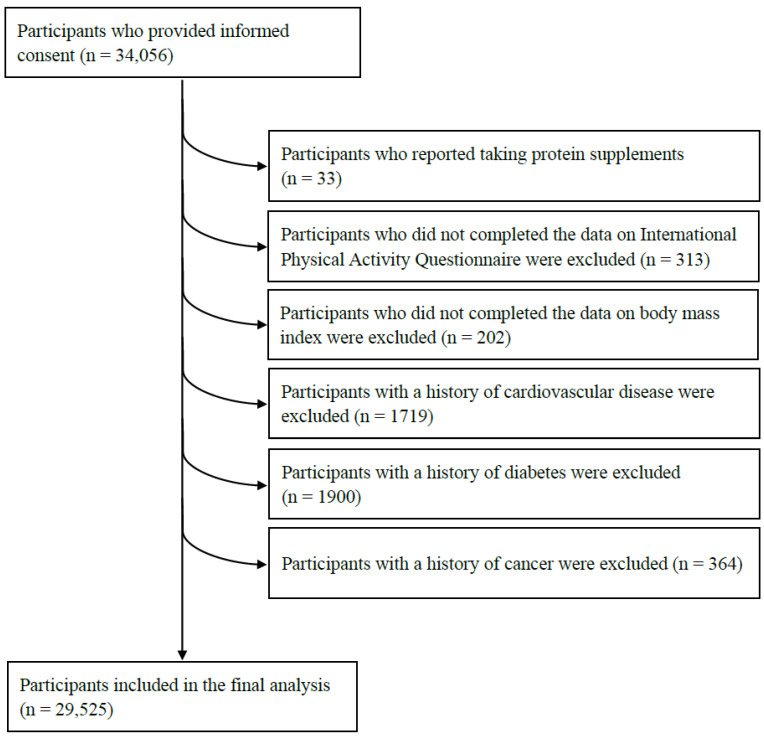
Flow chart describing the selection of participants for the current study.

In the present study, participants who were younger than 18 years, pregnant women, and those with muscle weakness caused by disease, such as disuse or motor neuron loss, were not recruited. The TCLSIH dataset for this population-based study recruited 34,056 subjects aged from 20.0 to 90.3 years. We included eligible adults who underwent handgrip strength (HGS) measurement and completed the diet questionnaire. We firstly excluded participants who were taking protein supplements (n = 33) or with missing data for any of the study variables (n = 515). We further excluded participants with serious diseases such as cardiovascular disease (CVD) (n = 1719), diabetes (n = 1900) and cancer (n = 364) because subjects with these diseases were more likely to develop sarcopenia, and their lifestyle habits may have changed after being diagnosed. After the exclusions, the final sample consisted of 29,525 participants (mean age: 41.6 years; 16,933 (53.8%) males). A flowchart is presented in Figure 1.

### 2.2. Handgrip Strength Measurement

Handgrip strength (HGS) was chosen as a reliable indicator of overall muscle strength because it is inexpensive, and easily measured both in clinical settings and in large epidemiological studies [26]. HGS was assessed, using a hand dynamometer (EH101; CAMRY, Guangdong, China), by a trained technician according to the instructions on the dynamometer. The interviewers showed the testing procedure to participants before the measurement trials. The procedure involved standing upright with their arms vertical, and squeezing the handle as strongly as they could for several seconds. A HGS test was performed two times for both hands, and the highest value was used for the analysis.

### 2.3. Dietary Assessment

Usually dietary intake over the past month was collected by the interviewers using a 100-item food frequency questionnaire (FFQ) with specified serving sizes. Participants indicated how often they had consumed each item by selecting from seven predefined responses ranging from “never” to “≥2 times per day” for foods, and eight responses ranging from “never” to “≥4 times per day” for beverages. The FFQ obtained information on soy food consumption by asking participants “In the past week/month, how many times of soy food (e.g., tofu, tofu sticks, soy milk, soy drink, and dried soybeans) did you have?” The reproducibility and validity of the FFQ have been evaluated among 150 subjects, randomly selected from the TCLSIH cohort study [9,27]. According to the validation study, the portion size of soy foods was 60.0 g/serving and 50 g/serving for men and women, respectively. The 2009 China Food Composition Tables were used to assess the nutrient intakes and daily energy intakes [28]. The quantity of each food consumed was calculated by multiplying a portion size assigned for each specific food by the amount consumed.

### 2.4. Measurement of Covariates

Covariates were chosen based on previous literature evidence on the potential determinants of muscle health and their associations with soy food consumption. Information on potential confounders of the study associations was derived from a standardized questionnaire. Self-reported information was obtained for sex, age, educational or professional qualification, monthly family income, tobacco use, frequency of alcohol consumption, medical conditions and family history of diseases. Total physical activity during the last week was evaluated using the short form of the International Physical Activity Questionnaire [29].

## 3. Statistical Analysis

Participants characteristics according to the soy food consumption categories in terms of relative (%) frequency for categorical variables and mean ± SD or medians (P25, P75) for continuous variables.

Analysis of covariance were used to calculate the least square means (LSM) and 95% confidence interval (CI) for HGS across the soy food consumption (with less than one time per week consumption as the reference category = 1). Except for the crude model, three models were further built with the aim of progressively assessing the confounding effect of the following blocks of covariates: model 1: adjusted for age, sex and BMI; model 2: previous model adjusted for lifestyle behaviors and disease conditions; model 3: previous model adjusted for total energy intake and dietary pattern.

Furthermore, the likelihood ratio test was used to explore the possible interactions between soy food consumption and covariates in the association with HGS. Stratified analyses which aimed to identify potential moderators of the study association were also performed. We conducted analyses stratified by sex, age, BMI and physical activity. We also performed sensitivity analyses with the energy-adjusted soy food consumption (grams/1000 kcal per day) instead of soy food consumption frequency.

Finally, we performed multiple logistic regression to examine the association between soy food consumption and low HGS. Low HGS was defined using the revised Asian Working Group for Sarcopenia (AWGS) 2019 as HGS lower than 28 kg for men and 18 kg for women [30].

All statistical analyses were conducted using SAS software (version 9.3; SAS Institute Inc., Cary, NC, USA), and *p* < 0.05 was considered to indicate statistical significance.

## 4. Results

A total of 29,525 participants [15,504 (52.5%) males] aged 40.9 (11.7) years were included in the final analysis. The mean (SD) HGS was 43.8 (7.1) kg for men, and 25.8 (4.8) kg for women, respectively. Table 1 presents the main baseline characteristics of all included participants, and according to categories of soy food consumption.

The relationships between the soy food consumption categories and HGS were presented in Table 2. Among the 29,525 adults, 5848 (19.8%) reported consuming soy food less than one time per week, 7220 (24.5%) consumed 1 time per week, 10,354 (35.1%) consumed 2–3 times per week and 6103 (20.7%) consumed ≥ 4 times per week. In the final adjusted models, soy food consumption categories were positively associated with HGS. The LSM and 95% CI for HGS, across soy food consumption categories, were 35.5 (34.2, 37.1) kg for <1 time per week, 36.1 (34.6, 37.6) kg for 1 time per week, 36.3 (34.8, 37.8) kg for 2–3 times per week, and 36.6 (35.1, 38.0) kg for ≥4 times per week *(p* for trend < 0.001).

We further explored associations between soy food consumption and HGS by sex, age, BMI categories and physical activity categories (Table 3). Through the stratified analyses, we found significant associations of soy food consumption with HGS in both sexes, in participants aged < 60 years, BMI ≥ 24 kg/m^2^, and PA ≥ 23.0 MET × hour/week. In the interaction analysis, we found that the soy food consumption and age and BMI have an interactive effect on HGS (both *p* < 0.001). It is worth noting that in participants age < 60 years or with BMI ≥ 24 kg/m^2^, soy food consumption has a stronger protective effect on HGS.

We observed similar results when energy-adjusted soy food consumption was used, instead of soy food consumption frequency (Table 4). The LSM (95% CI) for HGS across energy-adjusted soy food consumption categories were 35.6 (34.6, 37.5) kg for <5.81 g/kcal/day, 36.0 (34.7, 37.7) kg for 5.81–13.1 g/kcal/day, 36.3 (34.8, 37.8) kg for 13.1–20.2 g/kcal/day, and 36.3 (34.8, 37.7) kg for >20.2 g/kcal/day (*p* for trend < 0.001).

The relationships between the soy food consumption categories and low HGS were shown in Table 5. Compared with soy food consumption category less than one time per week, weekly consumption of ≥4 times significantly reduced the risk (36%) of low HGS. The odds ratio and 95% CI for low HGS across soy food consumption categories were 1.00 (reference) for <1 time per week, 0.944 (0.756, 1.178) for 1 time per week, 0.819 (0.662, 1.015) for 2–3 times per week, and 0.638 (0.485, 0.836) for ≥4 times per week (*p* for trend < 0.01).

## 5. Discussion

In this large-scale epidemiological study, we analyzed the association between soy food consumption and HGS in general Chinese adults. The main findings were that habitual soy food consumption was positively associated with HGS, after adjusting for potential confounding factors. Notably, the highest category of soy food consumption (≥4 times per week) was associated with a 1.1 kg increased HGS compared to the lowest category of soy food consumption (<1 time per week). In addition, compared with the soy food consumption category less than one time per week, weekly consumption ≥ 4 times was associated with a 36% (95% CI 16%, 51%) lower risk of low HGS. Our results highlight the potential role of soy food consumption as a healthy behavior to prevent muscle strength decline.

Soy foods are rich sources of protein and many other bioactive substances such as isoflavones, essential micronutrients, vitamins, monounsaturated and polyunsaturated fatty acids. Growing evidence have shown positive associations of soy food consumption and different health outcomes such as breast cancer, osteoporosis, cardiovascular disease, and menopausal symptoms [31,32,33,34]. However, to date, though most studies have revealed a protective effect of soy components (soy protein or soy isoflavone) on muscle health, related epidemiologic studies to determine the associations between soy food and muscle function were limited. Animal studies have demonstrated that soy protein or soy protein isolate ingestion stimulate muscle protein synthesis by activating the mTOR pathway and inhibiting FOXO transcription factors [35,36,37]. This protein anabolic effect was more pronounced in studies treated simultaneously with other nutrients or supplements, such as chromium, green tea, and leucine, thus indicating a synergic effect [38,39]. In addition, several RCTs have demonstrated that soy protein supplementation has a protective effect on muscle. A RCT conducted in sixteen healthy, young subjects by Reidy PT et al. demonstrated that dietary soy-dairy protein ingestion enhanced amino acid transporter mRNA expression and muscle protein synthesis [40]. Meta-analyses of nine RCTs have shown that the soy protein (a quintessential plant protein) plays a comparable role with animal proteins (whey protein) in promoting muscle strength gains [20]. Moreover, soy foods contain high levels of isoflavone. Messina S et al. found that five weeks of soy isoflavone supplementation blunted TNF-α, NF-κB, and MAPKs activation and ameliorated muscle function in mdx mice [41]. Similarly, it was suggested by Munguia L et al. that flavonoids could be used as a potential strategy for preventing muscle loss in adults [23]. Consistent with these studies, the present study found that a moderate (four times per week) consumption of soy food was significantly associated with an increased HGS (1.1 kg) compared to the low (<1 time per week) consumption of soy food in general Chinese adults. Our study supports the idea that soy food consumption might be recommended as a healthy behavior to prevent muscle strength decline.

Although the underlying mechanism is unclear, it is biologically plausible that higher soy food consumption has beneficial effects on HGS. One of the mechanisms by which soy food prevents muscle strength decline might be related to the soy protein synthesis effects. The intake of high levels of dietary protein is recognized as an effective strategy to prevent muscle decline in older adults. Older adults require more protein to stimulate muscle anabolism due to a decreased muscle protein synthesis [42]. In order to maintain metabolic homeostasis, older adults must consume more protein. Empirical evidence has indicated that diets based on the current Recommended Dietary Allowances (RDA) for protein intake (0.8/kg body weight (BW)/day) are not sufficient to maintain the nitrogen balance. A diet higher in protein (1.0–1.5 g/kg BW/day) than the RDA has been advocated by many researchers as a way to prevent or delay muscle atrophy due to aging [43]. Soy foods, such as soybeans are particularly abundant in protein, and are one of the highest vegetable sources of proteins. Therefore, it is rational that a diet high in soy food has a protective effect on muscle strength decline.

The other mechanism for the beneficial effects of soy food on muscle function might be the antioxidant effect of nutrients in soy food. Because oxidative stress is thought to contribute to muscle atrophy and the pathogenesis of sarcopenia, higher soy food consumption might decrease the risk of muscle decline through the strong radical-scavenging and anti-oxidative effects of these compounds. Soy foods and products are rich in biologically active compounds and nutrients, such as isoflavones, unsaturated fatty acids, zinc and vitamin E. Most of these compounds exert an antioxidant effect [1,15]. Therefore, soy food can attenuate oxidative stress, exerting protective effects on muscle.

A robust association has been established between HGS and adverse health outcomes in clinical settings due to its ease, simplicity, portability and affordability [44]. One meta-analysis found that a 1 kg increase in HGS reduced 5% risk of functional status in older adults [45] and reduced 3% risk of all-cause mortality in adults [46]. Moreover, another meta-analysis performed by Lopez-Bueno R et al. reported that the pooled odds ratio of ADL associated with a 1 kg decrease in HGS was 1.09 (95% CI: 1.05, 1.13) [47]. In our study, we found that the highest category of soy food consumption (≥4 times per week) was associated with a 1.1 kg reduced HGS compared to the lowest category of soy food consumption (<1 time per week). Furthermore, we found that weekly consumption of ≥4 times significantly reduced the risk (36%) of low HGS compared with the soy food consumption category <1 times per week. The significant difference in HGS between the lowest and highest soy food consumption indicated that high frequency consumption of soy food may have potentially beneficial effects on muscle health, consequently improving physical function, reducing disability and decreasing risk of death.

There were several important strengths in our study. Firstly, in this relatively large sample size population study, detailed lifestyle and diet information were collected and we controlled for a variety of possible confounding factors. Additionally and importantly, our results added to the limited data on the associations between soy food consumption and muscle strength decline. Age-related muscle strength decline is a significant component of sarcopenia. In addition, sarcopenia can occur in adults, but is more prevalent among the older population. Due to its association with a greater risk for disability, morbidity and mortality, sarcopenia is considered a significant public health problem [48,49]. Hence, our findings have important implications for preventing and avoiding sarcopenia by increasing dietary soy food consumption. However, besides these strengths, several limitations should also be noted. First, the cross-sectional design precluded the determination of causality between soy food consumption and HGS. In addition, although our analyses were adjusted for relevant covariates, unknown or inaccurately measured confounders cannot be excluded.

In conclusion, higher habitual soy food consumption was positively associated with HGS in general Chinese adults. This indicated that a diet high in soy food might have a protective effect on muscle strength decline. Given that soy food is frequently consumed in China, our findings have important public health implications for preventing muscle strength decline.

## Figures and Tables

**Table 1:** Baseline characteristics of the participants according to frequency of soy food consumption (n = 29,525).

Characteristics ^a^	Frequency of Soy Food Consumption				*p* for Trend ^b^
	<1 Time/Week	1 Time/Week	2–3 Times/Week	≥4 Times/Week	
No. of participants	(n = 5848)	(n = 7220)	(n = 10,354)	(n = 6103)	-
Sex (men, %)	51.0	53.5	53.7	50.9	0.91
Age (years)	40.6 ± 11.4	40.0 ± 11.1	40.9 ± 11.7	42.1 ± 12.4	0.17
BMI (kg/m^2^)	34.7 ± 10.9	35.4 ± 10.9	35.6 ± 10.8	35.0 ± 10.7	0.05
WC (cm)	24.5 ± 3.75	24.5 ± 3.70	24.6 ± 3.80	24.6 ± 3.70	0.11
PA (METs × hour/week)	82.9 ± 11.1	82.8 ± 11.1	83.2 ± 11.1	83.1 ± 11.1	0.05
Total energy intake (kcal/day)	1817.1 (1394.9, 2226.8)	1999.7 (1638.5, 2336.1)	2168.1 (1851.3, 2446.7)	2279.3 (2023.4, 2581.6)	<0.0001
“Healthy” dietary pattern score	−0.57 (−0.96, −0.05)	−0.40 (−0.78, 0.07)	−0.08 (−0.48, 0.42)	0.47 (−0.05, 1.16)	<0.0001
“Fruits and sweet” dietary pattern score	−0.32 (−0.67, 0.10)	−0.19 (−0.54, 0.26)	−0.07 (−0.47, 0.41)	0.06 (−0.45, 0.72)	<0.0001
“Animal foods” dietary pattern score	−0.24 (−0.52, 0.14)	−0.17 (−0.47, 0.28)	−0.20 (−0.52, 0.27)	−0.29 (−0.68, 0.27)	<0.0001
TC (mmol/L)	4.77 ± 0.90	4.74 ± 0.89	4.74 ± 0.89	4.76 ± 0.92	<0.05
TG (mmol/L)	1.08 (0.76, 1.67)	1.09 (0.76, 1.6)	1.07 (0.76, 1.62)	1.07 (0.75, 1.62)	0.10
LDL-C (mmol/L)	2.82 ± 0.80	2.79 ± 0.79	2.78 ± 0.79	2.78 ± 0.81	0.001
HDL-C (mmol/L)	1.38 ± 0.38	1.38 ± 0.37	1.38 ± 0.38	1.40 ± 0.38	0.41
FBG (mmol/L)	5.02 ± 0.47	4.99 ± 0.47	5.00 ± 0.48	5.01 ± 0.49	0.11
SBP (mmHg)	120.4 ± 15.8	120.2 ± 15.8	121.1 ± 16.2	121.1 ± 16.4	0.08
DBP (mmHg)	76.2 ± 11.4	76.1 ± 11.4	76.6 ± 11.5	76.5 ± 11.6	0.06
Smoking status (%)					
Current smoker	21.0	21.1	20.0	18.9	<0.001
Ex-smoker	4.82	4.81	5.49	5.73	<0.001
Non-smoker	74.2	74.1	74.5	75.4	0.12
Drinking status (%)					
Everyday drinker	4.67	4.37	4.76	4.98	0.25
Sometime drinker	53.7	57.2	57.7	55.4	0.04
Ex-drinker	10.6	8.56	9.29	8.94	<0.05
Non-drinker	31.0	29.9	28.3	30.7	0.21
Educational level (≥college grade, %)	65.2	69.4	68.5	69.0	<0.001
Employment status (%)					
Managers	40.6	42.5	43.1	46.0	<0.0001
Professionals	15.9	17.1	17.9	15.9	0.57
Other	43.5	40.4	39.0	38.2	<0.0001
Household income (≥10,000 Yuan, %)	34.1	39.0	40.1	40.3	<0.0001
Hypertension (%)	21.4	20.7	22.6	23.0	<0.001
Hyperlipidemia (%)	44.9	43.3	43.5	43.6	0.47
Family history of diseases (%)					
CVD	28.3	28.2	29.3	32.0	<0.0001
Hypertension	48.7	48.8	50.4	50.6	<0.01
Diabetes	23.5	23.0	24.6	25.0	<0.05

^a^ Continuous variables are expressed as mean ± SD or medians (P25, P75) and categorical variables are expressed as percentages. BMI, body mass index; CVD, cardiovascular disease; DBP, diastolic blood pressure; FBG, fasting blood glucose; HDL-C, high-density lipoprotein cholesterol; LDL-C, low-density lipoprotein cholesterol; METs, metabolic equivalents; PA, physical activity; SBP, systolic blood pressure; TC, total cholesterol; TG, triglycerides; WC, waist circumference. ^b^ *p* values were calculated using analysis of variance or logistic regression analysis.

**Table 2:** The association between frequency of soy food consumption and handgrip strength (n = 29,525).

	Frequency of Soy Food Consumption				*p* for Trend ^a^
	<1 Time/Week	1 Time/Week	2–3 Times/Week	≥4 Times/Week	
	(n = 5848)	(n = 7220)	(n = 10,354)	(n = 6103)	
Crude	34.5 (34.3, 34.8) ^b^	35.3 (35.1, 35.6)	35.6 (35.4, 35.8)	35.3 (35.0, 35.6)	<0.0001
Model 1 ^c^	34.4 (34.2, 34.5)	34.7 (34.5, 34.8)	34.9 (34.8, 35.0)	35.2 (35.1, 35.4)	<0.0001
Model 2 ^d^	34.4 (34.3, 34.6)	34.7 (34.6, 34.8)	34.9 (34.8, 35.0)	35.2 (35.1, 35.4)	<0.0001
Model 3 ^e^	35.5 (34.2, 37.1)	36.1 (34.6, 37.6)	36.3 (34.8, 37.8)	36.6 (35.1, 38.0)	<0.0001

^a^ Analysis of covariance. ^b^ Adjusted least square mean (95% confidence interval) (all such values). ^c^ Adjusted for age, sex and body mass index. ^d^ Additionally adjusted for smoking status, alcohol drinking status, educational level, occupation, monthly family income, physical activity, hypertension, hyperlipidemia and family history of disease (including cardiovascular disease, hypertension, hyperlipidemia and diabetes). ^e^ Additionally adjusted for total energy intake and dietary pattern.

**Table 3:** Stratified analysis of associations between soy food intake and handgrip strength (n = 29,525).

	Frequency of Soy Food Consumption				*p* for Trend ^a^	*p* for Interaction
	<1 Time/Week	1 Time/Week	2–3 Times/Week	≥4 Times/Week		
Sex						
Men (n = 15,504)	43.4 (43.2, 43.7) ^b^	43.7 (43.5, 43.9)	43.8 (43.7, 44.0)	44.1 (43.9, 44.4)	<0.01	0.10
Women (n = 14,021)	25.4 (25.2, 25.5)	25.6 (25.4, 25.8)	25.9 (25.8, 26.1)	26.3 (26.1, 26.5)	<0.0001	
Age (year)						
<60 (n = 27,248)	35.1 (34.9, 35.3)	35.4 (35.3, 35.5)	35.6 (35.5, 35.8)	36.0 (35.8, 36.1)	<0.001	<0.0001
≥60 (n = 2277)	31.7 (30.9, 32.0)	31.8 (31.1, 32.2)	31.8 (31.4, 32.1)	31.9 (31.5, 32.4)	0.19	
Body mass index (kg/m^2^)						
<24 (n = 13,610)	30.8 (30.4, 31.0)	30.9 (30.7, 31.1)	31.0 (30.9, 31.2)	31.2 (31.0, 31.4)	0.35	<0.001
≥24 (n = 15,915)	38.5 (38.2, 38.7)	38.7 (38.5, 38.9)	39.0 (38.9, 39.2)	39.3 (39.1, 39.5)	<0.001	
Physical activity (MET × hour/week)						
<23.0 (n = 20,941)	34.8 (34.5, 35.0)	34.8 (34.6, 35.0)	34.9 (34.7, 35.0)	35.1 (34.9, 35.2)	0.13	0.22
≥23.0 (n = 8584)	36.1 (35.8, 36.5)	36.3 (36.0, 36.5)	36.5 (36.3, 36.7)	37.0 (36.8, 37.3)	<0.0001	

^a^ Analysis of covariance. Adjusted for age, sex, body mass index, smoking status, alcohol drinking status, educational level, occupation, monthly family income, physical activity, hypertension, hyperlipidemia, and family history of disease (including cardiovascular disease, hypertension, hyperlipidemia, and diabetes), total energy intake, and dietary pattern. ^b^ Adjusted least square mean (95% confidence interval) (all such values).

**Table 4:** Association of energy-adjusted soy food consumption with handgrip strength (n = 29,525).

	Categories of Energy-Adjusted Soy Food Consumption (g/kcal/Day)				*p* for Trend ^a^
	<5.81	5.81–13.1	13.1–20.2	>20.2	
Participants, n	(n = 7383)	(n = 7381)	(n = 7381)	(n = 7380)	
Crude	34.7 (34.5, 35.0) ^b^	35.4 (35.2, 35.7)	35.6 (35.4, 35.8)	35.0 (34.7, 35.3)	<0.0001
Model 1 ^c^	34.6 (34.4, 34.7)	34.8 (34.6, 34.9)	34.9 (34.8, 35.0)	35.2 (34.8, 35.4)	<0.0001
Model 2 ^d^	34.6 (34.5, 34.8)	34.8 (34.7, 34.9)	34.9 (34.8, 35.0)	35.2 (34.8, 35.4)	<0.001
Model 3 ^e^	35.6 (34.6, 37.5)	36.0 (34.7, 37.7)	36.3 (34.8, 37.8)	36.3 (34.8, 37.7)	<0.001

^a^ Analysis of covariance. ^b^ Adjusted least square mean (95% confidence interval) (all such values). ^c^ Adjusted for age, sex, and body mass index. ^d^ Additionally adjusted for smoking status, alcohol drinking status, educational level, occupation, monthly family income, physical activity, hypertension, hyperlipidemia, and family history of disease (including cardiovascular disease, hypertension, hyperlipidemia, and diabetes). ^e^ Additionally adjusted for total energy intake, and dietary pattern.

**Table 5:** The association between soy food consumption and low handgrip strength (n = 29,525).

	Frequency of Soy Food Consumption				*p* for Trend ^a^
	<1 Time/Week	1 Time/Week	2–3 Times/Week	≥4 Times/Week	
Crude	1.00 (reference)	0.904 (0.736, 1.112) ^b^	0.765 (0.628, 0.932)	0.668 (0.529, 0.842)	<0.01
Age, sex and body mass index-adjusted model	1.00 (reference)	0.954 (0.773, 1.177)	0.762 (0.624, 0.932)	0.601 (0.474, 0.761)	<0.0001
Multivariable model ^c^	1.00 (reference)	0.944 (0.756, 1.178)	0.819 (0.662, 1.015)	0.638 (0.485, 0.836)	<0.01

^a^ Multiple logistic regression analysis. ^b^ Odds ratios (95% confidence interval) (all such values). ^c^ Adjusted for age, sex, body mass index, smoking status, drinking status, education levels, employment status, household income, physical activity, hypertension, hyperlipidemia and family history of diseases (cardiovascular disease, hypertension, hyperlipidemia and diabetes), total energy intake and dietary pattern.

## Data Availability

Upon reasonable request, data can be obtained from the corresponding author.
